# Single-cell RNA sequencing reveals cellular and molecular landscape of fetal cystic hygroma

**DOI:** 10.1186/s12920-024-01859-x

**Published:** 2024-04-22

**Authors:** Fang Fu, Xin Yang, Ru Li, Yingsi Li, Hang Zhou, Ken Cheng, Ruibin Huang, You Wang, Fei Guo, Lina Zhang, Min Pan, Jin Han, Li Zhen, Lushan Li, Tingying Lei, Dongzhi Li, Can Liao

**Affiliations:** Department of Prenatal Diagnostic Centre, Guangzhou Women and Children’s Medical Centre, Guangzhou Medical University, 510623 Guangzhou, China

**Keywords:** Single-cell RNA-sequencing, Lymphatic endothelial cells, Cystic hygroma, Vascular endothelial growth factor

## Abstract

**Background:**

The molecular mechanism of fetal cystic hygroma (CH) is still unclear, and no study has previously reported the transcriptome changes of single cells in CH. In this study, single-cell transcriptome sequencing (scRNA-seq) was used to investigate the characteristics of cell subsets in the lesion tissues of CH patients.

**Methods:**

Lymphoid tissue collected from CH patients and control donors for scRNA-seq analysis. Differentially expressed gene enrichment in major cell subpopulations as well as cell-cell communication were analyzed. At the same time, the expression and interactions of important VEGF signaling pathway molecules were analyzed, and potential transcription factors that could bind to *KDR* (*VEGFR2*) were predicted.

**Results:**

The results of scRNA-seq showed that fibroblasts accounted for the largest proportion in the lymphatic lesions of CH patients. There was a significant increase in the proportion of lymphatic endothelial cell subsets between the cases and controls. The VEGF signaling pathway is enriched in lymphatic endothelial cells and participates in the regulation of cell-cell communication between lymphatic endothelial cells and other cells. The key regulatory gene *KDR* in the VEGF signaling pathway is highly expressed in CH patients and interacts with other differentially expressed *EDN1*, *TAGLN*, and *CLDN5* Finally, we found that STAT1 could bind to the *KDR* promoter region, which may play an important role in promoting *KDR* up-regulation.

**Conclusion:**

Our comprehensive delineation of the cellular composition in tumor tissues of CH patients using single-cell RNA-sequencing identified the enrichment of lymphatic endothelial cells in CH and highlighted the activation of the VEGF signaling pathway in lymphoid endothelial cells as a potential modulator.

**Simple summary:**

The molecular and cellular pathogenesis of fetal cystic hygroma (CH) remains largely unknown. This study examined the distribution and gene expression signature of each cell subpopulation and the possible role of VEGF signaling in lymphatic endothelial cells in regulating the progression of CH by single-cell transcriptome sequencing. The enrichment of lymphatic endothelial cells in CH and the activation of the VEGF signaling pathway in lymphatic endothelial cells provide some clues to the pathogenesis of CH from the perspective of cell subpopulations.

**Supplementary Information:**

The online version contains supplementary material available at 10.1186/s12920-024-01859-x.

## Introduction

Fetal cystic hygroma (CH) is a developmental abnormality caused by an abnormal interaction between the lymphatic system and the venous system and is a type of lymphatic system malformation [1]. CH can result in single or multiple large cystic lesions that can occur in any part of the body. Lymphatic vessels play an important role in many physiological and pathological processes in the human body, therefore, when CH occurs in lymphatic vessels, it can lead to the inability of lymphatic vessels to communicate with the normal jugular vein, which is associated with adverse pregnancy outcomes [2]. CH has been reported in about 1 in 800 pregnancies and 1 in 8000 live births. Although with the development of next-generation sequencing technology, genetic variants such as *SOX9*, *KDR*, and *BRCA1* are involved in the pathogenesis of cystic hygroma, the specific pathogenesis of cystic hygroma remains unclear [3–6]. There are currently no studies that explain the pathogenesis of CH from the perspective of single cell subpopulations.

Single-cell transcriptome sequencing (scRNA-seq) is one of the effective methods to analyze and solve the heterogeneity of complex biological systems. It includes four steps: single cell isolation, reverse transcription, cDNA amplification, and sequencing library construction, which can accurately determine the cell type, gene expression signature, and regulatory network analysis [7]. Currently, single-cell sequencing has been applied to reveal the characteristics and heterogeneity of cell subsets in a variety of diseases such as tumors [8]. It is worth noting that scRNA-seq also promotes the understanding of lymphatic-vascular system related diseases. In the lymph-vascular system, lymphatic endothelial cells (LECs) are distributed in lymphatic vessels and play a central role in the immune response [9]. Recent studies based on scRNA-seq have found that homeobox d8, T-box 1, and ETS transcription factor 3 are involved in the regulation of LEC development [10]. Furthermore, transcriptome profiling of LECs in mouse cutaneous lymph nodes identified different LEC subsets and predicted their functions [11]. Recently, it has been found that LECs may play an important role in the pathological process of CH [12]. However, studies on cell subsets and transcriptome profiling of lesion tissues from CH patients are still lacking.

In this study, we performed scRNA-seq on tumor samples from CH patients to analyze the distribution and gene expression signature of each cell subpopulation and to assess the possible role of VEGF signaling in lymphatic endothelial cells in regulating the progression of CH. This study hopes to provide a cellular explanation for the pathogenic mechanism of CH and provide prospects for the development of drugs that may target VEGF signaling pathway molecules at the single-cell level.

## Materials and methods

### Patient samples

Two cohorts of CH fetal cases and normal controls who terminated pregnancy at 11–13 + 6 weeks of early pregnancy enrolled in this study (Table 1). In all CH fetal cases, cystic hygroma was detected by prenatal ultrasonography and had negative results for chromosomal and Mendelian monogenic conditions by CMA (Affymetrix CytoScanHD array) and high-coverage WES (> 200-fold), before scRNA-seq was performed. Negative controls consisted of healthy fetuses voluntarily aborted by the pregnant woman. All enrolled cases and controls underwent dissection of lymphoid tissue from the neck for scRNA-seq analysis.

**Table 1:** Detailed clinical information of patient samples

Patent	Gestational age (weeks)	Diagnostic results by ultrasonography	chromosome examination results
1	14+	Thickening of the neck skin, small jaw, and inversion of the right foot	unknown
2	14+	Fetal cervical cystic hygroma, Single umbilical artery	unknown
3	12+	Fetal cervical cystic hygroma	unknown
4	12+	Fetal cystic hygroma	unknown
5	12+	No abnormalities	46, XN
6	14+	No abnormalities	46, XN
7	14+	No abnormalities	46, XN
8	10+	No abnormalities	46, XN
9	12+	No abnormalities	46, XN
10	9+	No abnormalities	46, XN

All procedures were conducted following the Declaration of Helsinki. The collection protocol of lymphoid tissue from CH patients and corresponding normal control patients was approved by Guangzhou Women and Children’s Medical Center Hospital (**Protocol # 2,018,021,402**). All subjects gave written, informed consent before participating.

### Tissue dissociation and preparation of single-cell suspensions

Collected samples were immediately placed in an ice-cold preservation solution and then transported to the laboratory to maintain viability. After being mechanically dissected into 1- to 2-mm small pieces, the tissue fragments were enzymatically dissociated in 10 ml of solution containing 2 mg/ml collagenase type I, 1 mg/ml dispase II, and 1 unit/ml DNase I in PBS with 1%FBS for 30 min by gentle stirring 6 times in a 37 °C water bath. Subsequently, the disaggregated tissue components were filtered through a 70-µm cell strainer and lysed with 1X RBC lysis buffer to remove red blood cells. The cell pellets were washed twice in PBS (Life Technologies) + 0.04% BSA (Sigma) and re-suspended in PBS + 0.04% BSA. Sample viability was assessed via Trypan Blue (ThermoFisher) and using an automatic cell counter (Countstar).

### 10× Genomics single-cell RNA sequencing

Droplet-based scRNA-seq libraries were prepared as outlined by the 10x Genomics Single Cell 3′ v3 Reagent Kit user guide. Briefly, cells were loaded onto the 10x Genomics single-cell-A chip. After droplet generation, samples were transferred onto a pre-chilled 8-well tube (Eppendorf), heat-sealed and reverse transcription was performed using a Veriti 96-well thermal cycler (Thermo Fisher). After the reverse transcription, cDNA was recovered using Recovery Agent provided by 10x followed by a Silane DynaBead clean-up (Thermo Fisher) as outlined in the user guide. Purified cDNA was amplified for 12 cycles before being cleaned up using SPRIselect beads (Beckman). Samples were diluted 4:1 and run on a Bioanalyzer (Agilent Technologies) to determine cDNA concentration. cDNA libraries were prepared as outlined by the Single Cell 3′Reagent Kits v3 user guide with appropriate modifications to the PCR cycles based on the calculated cDNA concentration (as recommended by 10X Genomics).

The molarity of each library was calculated based on library size as measured using a bioanalyzer (Agilent Technologies) and qPCR amplification data. Samples were pooled and normalized to 10 nM, then diluted to 2 nM using elution buffer with 0.1% Tween20 (Sigma). Samples were sequenced by a Novaseq 6000 machine with 150-bp paired-end reads.

### Unsupervised clustering of cells and uniform manifold approximation and projection (UMAP) visualization

The analysis of single-cell sequencing dataset was processed as described previously [13–15]. Raw sequencing reads were aligned to the human genome reference sequence (GRCh38). The CellRanger (v3.1.0, 10X Genomics) analysis pipeline was used to generate a digital gene expression matrix from this data. The raw digital gene expression matrix (UMI counts per gene per cell) was filtered, normalized, and clustered using R (version 4.1.0). Cell and gene filtering was performed as follows: Cells that had fewer than 500 detected genes, or greater than 10,000 UMIs, as well as cells that contained greater than 10% of reads from mitochondrial genes were removed. Genes detected (UMI count > 0) in less than three cells were removed. After filtering, a total of 81,849 cells were left for the following analysis. After principal component analysis (PCA), the first 30 principal. components were selected for clustering the cells using standard package procedures. The “ggplot” was used for the visualization of PCA. A resolution of 0.5 was used with uniform manifold approximation and projection (UMAP) analysis visualization. With consideration of the expression of specific gene markers, 15 cell types were identified and only endothelial cells were pooled for downstream analysis (Table 2).

**Table 2:** Gene signatures of 15 cells

Type	Cell cluster	Gene signatures
1	fibroblast	MME, CD121, ITGB1, CD47, CD81,LRP1
2	keratinocyte progenitor cell	CD34, TP63
3	astrocyte	ALDH1L1, GFAP, NFIA, S100B
4	perithelial cell	PDGFRB, CSPG4, CD34
5	vascular progenitor cell	KIT, MKI67
6	endothelial progenitor cell	FLT3, PECAM1, CD34, THY1
7	circulating progenitor cell	ALDH, PROM1, CD14, MCAM, CD34
8	erythrocyte	GYPA, PTPRC, ITGAV, ITGB3
9	macrophage	CD163, MRC1, FCGR1A
10	B/T lymphocyte cells	CD3, CD19, CCR6
11	smooth muscle cell	ACTA1, DES
12	dendritic cell	ITGAX, CD209, CD83
13	keratinocyte	ALDH, CD44, TFRC, ITGA6
14	hematopoietic stem cell	PROM1, CD34, KIT
15	lymphatic endothelial cell	PECAM1, CD34, PDPN, PROX1, FLT4

### Identification of differentially expressed genes (DEGs)

Differential expression analysis comparing the CH versus control samples was performed with “cellranger”., *p* <. Deferentially expressed genes were calculated with fold-change (logFC > 1.5, FDR-adjusted p< 0.05). Genes were expressed in more than 25% of the cells.

### Kyoto encyclopedia of genes and genomes (KEGG) analysis

Evaluation of lymphatic endothelial cell status by KEGG analysis of differential gene enrichment in lymphatic endothelial cells (KEGG terms with p-value < 0.05 were selected for subsequent analysis). KEGG databases were used to analyze pathways enriched by R packages (version 4.1.0).

### Cell − cell interaction (CCI) analysis

Ligand-receptor (L-R) interactions and co-expression of a given interaction pair (int-pair) at the cell cluster level were assessed in cell-to-cell interactions. Visualize cell clusters, genes, and biological functions in cell-cell interactions with CellChat.

### Protein-protein interaction (PPI) network and hub gene identification

We constructed a PPI network of DEGs among VEGF pathway-related genes in lymphatic endothelial cells using the STRING database (https://string-db.org/ )to indicate functions and interactions between proteins. Cytoscape (https://cytoscape.org/) was used to further visualize the obtained PPI network [16]. Nodes in the PPI network represent proteins, and lines indicate interactions between them, and the node with the largest interaction is considered the hub gene. The degree algorithm was used to extract the hub genes in the PPI network.

### Transcription factor binding site prediction

For the KDR promoter DNA sequence ranging from − 2000 to + 200 bp, Use KnockTF analysis of transcription factor binding (http://www.licpathway.net/KnockTF/index.html).

### Statistical analysis

Variables between groups were compared by the Wilcoxon t-test. *P* < 0.05 was set as a significant difference in all statistical methods. R software version 4.1.0 (http://www.R-project.org) was used for data analysis and generation of figures, including “cellrangerRkit” (2.0.0), “Seurat” (version 3.1.1), “ClusterProfiler” (version 3.10.1), “org.Hs.eg.db” (version 3.13.0), “ggplot2” (version 3.1.0), “enrichplot” (version 1.12.3), “pheatmap”(1.0.12), “STRINGdb” (version 2.0.2).

## Results

### Transcripts were changed significantly in lymphatic endothelial cell subsets of cystic hygroma patients’ tissue

**Fig. 1:**
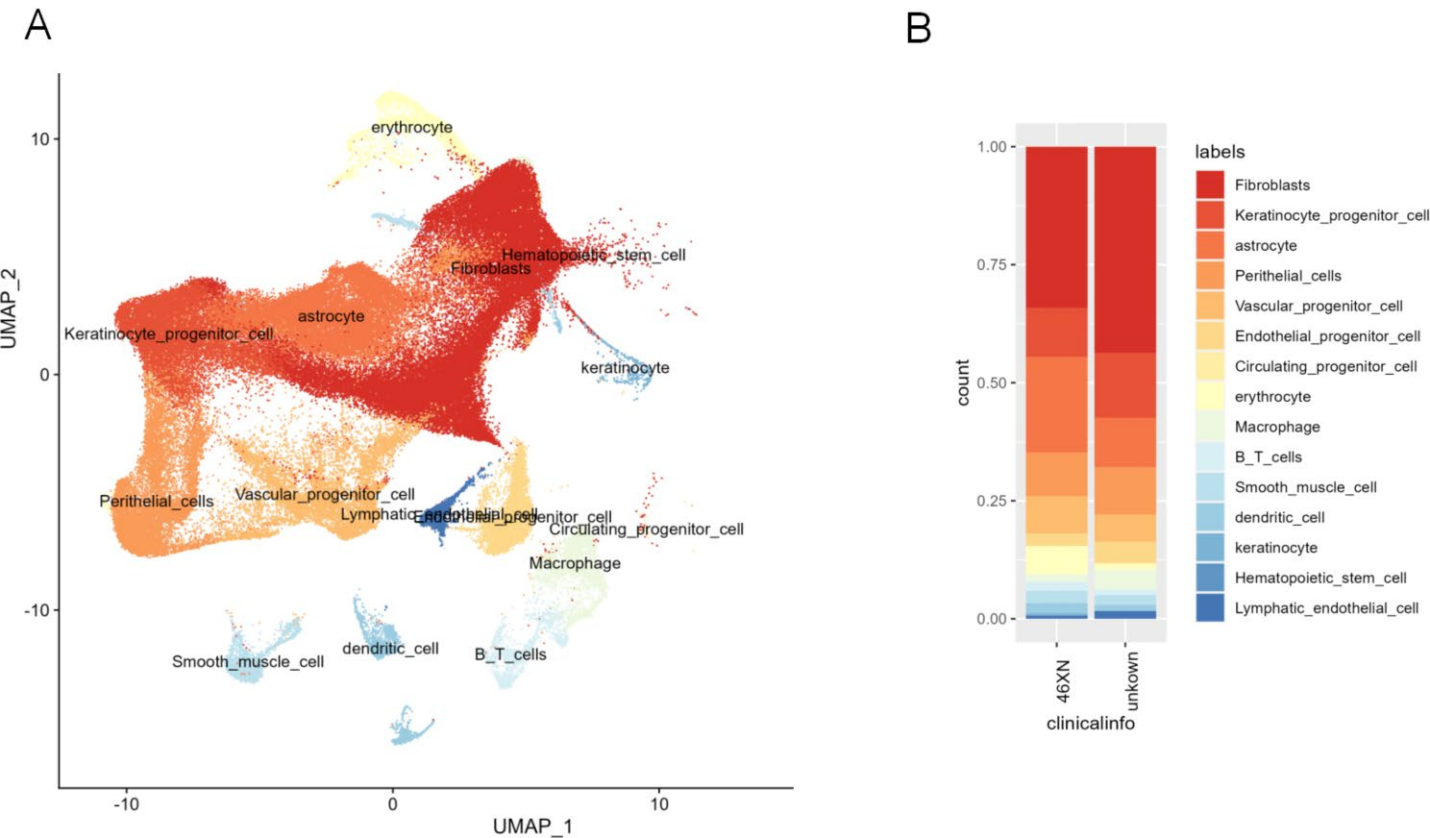
Single-cell RNA sequencing showing the transcriptome profile of tissue from fetal cystic hygroma patients and controls. (**A**) UMAP plot of all the single cells from the healthy control group and CH tissue reveals tumor-specific clusters. 500 cells were extracted randomly from each sample. (**B**) Stacked bar chart showing the proportion of differentially expressed genes in each tumor-specific cluster cell subpopulation between CH and control. CH, fetal cystic hygroma; UMAP, uniform manifold approximation and projection

To characterize the cellular and molecular features of lymphatic lesions in CH patients, single-cell transcriptome sequencing was performed on lymphatic tissues from cystic hygroma patients (*n* = 4) and controls (*n* = 6). By comparing the differences in gene transcripts 15 cell clusters (including fibroblast, keratinocyte progenitor cell, astrocyte, perithelial cell, vascular progenitor cell, endothelial progenitor cell, circulating progenitor cell, erythrocyte, macrophage, B/T lymphocyte cell, smooth muscle cell, dendritic cell, keratinocyte, hematopoietic stem cell, and lymphatic endothelial cell) between CH and controls, we found differences in the proportion of different cells in the lymphatic tissue. As shown in Fig. 1, fibroblasts accounted for the largest proportion in the lymphoid tissues of CH patients and normal controls while hematopoietic stem cells accounted for the smallest proportion (Fig. 1A&B). Lymphatic endothelial cells can be used as CH-specific markers to study the cell line development of cystic hygroma tumors [17]. Therefore, we also focused on the changes in lymphatic endothelial cell clusters and found that these cells were significantly upregulated in the CH group compared with the control group. Further analysis revealed that the lymphatic endothelial cell clusters were significantly elevated in CH patients compared with controls (Fig. 1B).

**Fig. 2:**
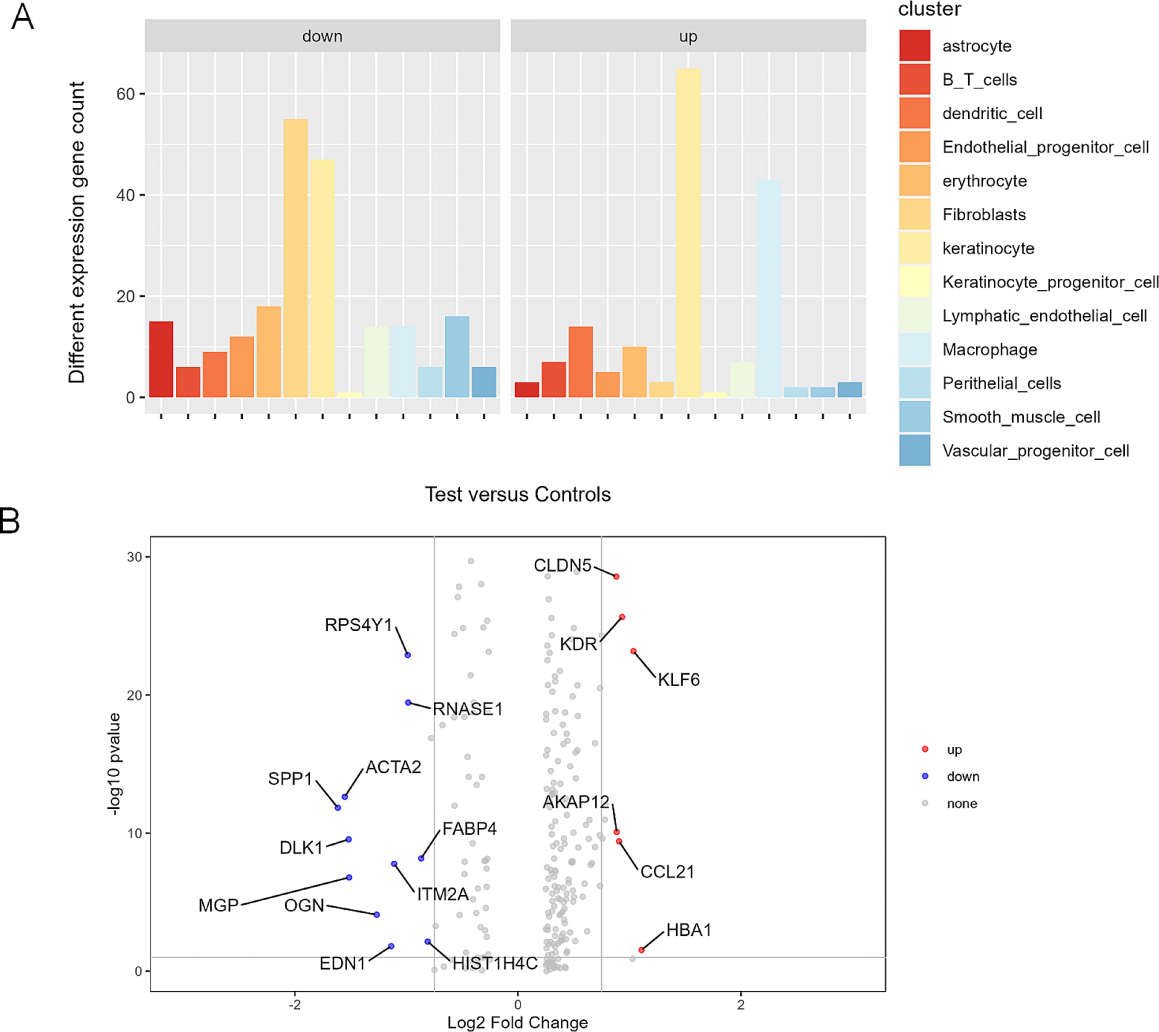
Transcript expression differences in lymphatic endothelial cell subsets in Cystic hygroma patients and controls. (**A**) Using FC > 2, p-value < 0.05 as the standard to analyze fetal cystic hygroma tissue and normal control tissue, it was found that different cell subgroups had corresponding differentially expressed genes. (**B**) The volcano plot shows the specific differential gene expression in lymphatic endothelial cells in CH compared with healthy controls

Further analysis of each cell subpopulation revealed that there were up-and down-regulated genes in each cell cluster in the CH group compared with the control group. As shown in Fig. 2A, compared with the control group, in the CH group, the two cell subpopulations with the most up-regulated genes were keratinocyte and macrophage, and the two cell subpopulations with the most down-regulated genes were fibroblasts and keratinocytes. Compared with the control group, the lymphatic endothelial cells in the CH group also had many up-regulated and down-regulated genes. The genes with differences in lymphatic endothelial cells are shown in Fig. 2B. The up-regulated genes are *CLDN5*, *KDR*, *KLF6*, *AKAP12*, *CCL21* and *HBA1*, and the down-regulated genes are *RPS4Y1*, *RNASE1*, *SPP1*, *ACTA2*, *DLK1*, *MGP*, *FABP4*, *OGN* and *ITM2A*, *END1* and *HIST1H4C*.

The above results showed that, compared with normal controls, the proportion of various types of cells in the lymphoid tissue of CH patients changed significantly, among which lymphatic endothelial cells had changes in gene expression.

### The VEGF signaling pathway is a hub for abnormalities of lymphatic endothelial cells in CH

**Fig. 3:**
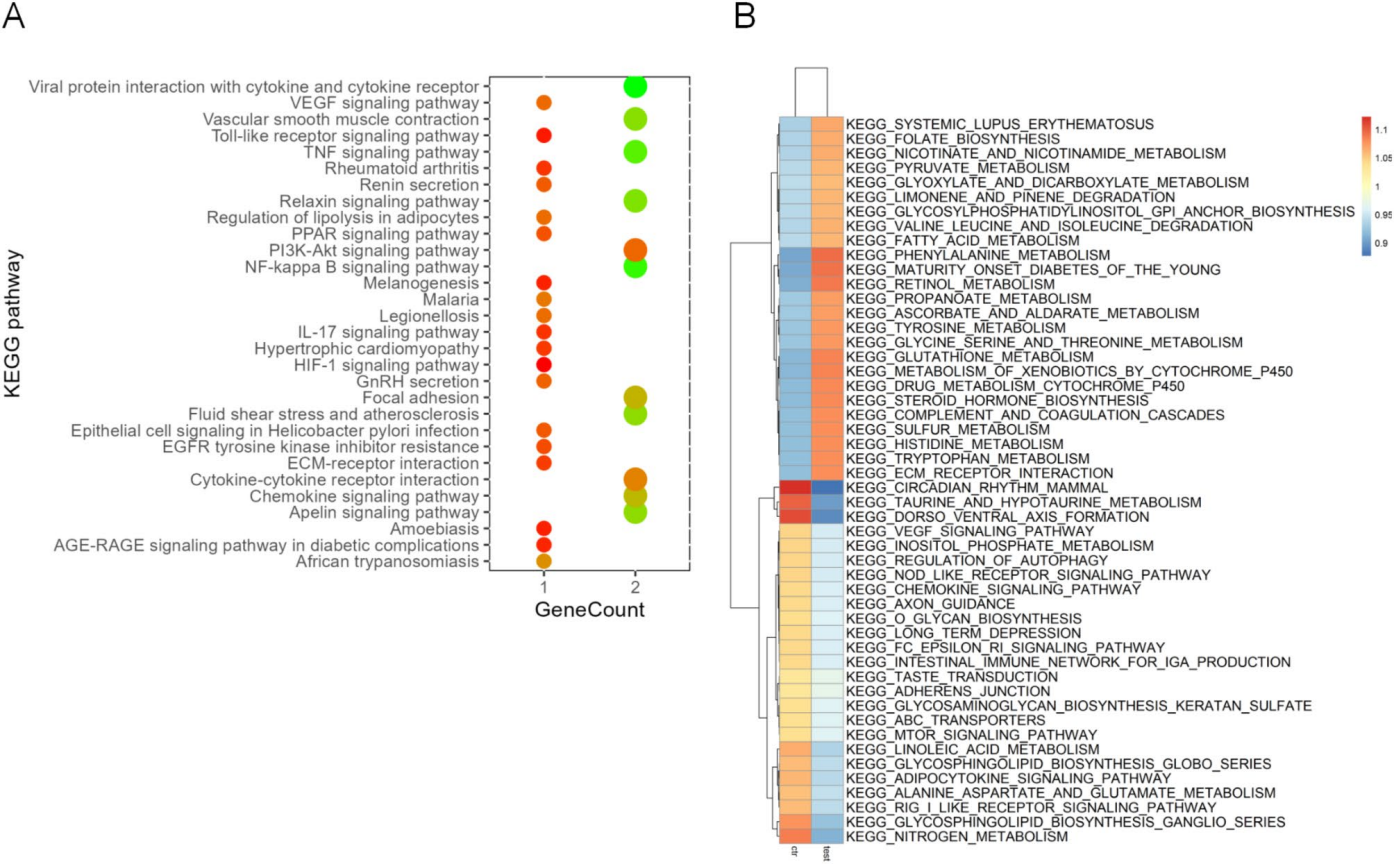
Lymphatic endothelial cells VEGF activity shapes the transcriptional landscape in CH. (**A**) The bubble plot shows KEGG enrichment analysis of all genes with significant differences in lymphatic endothelial cells of CH group compared with healthy controls; *p* < 0.05. (**B**) AUCell evaluates the cell function of lymphatic endothelial cell subsets in CH group and healthy control group and shows the top 50 KEGG signaling pathway enrichment in CH.

To predict the function of these differentially expressed genes in lymphatic endothelial cells, KEGG enrichment analysis was performed on these differentially expressed genes. The results showed that these differentially expressed genes were mainly enriched in viral protein interaction with cytokine and cytokine receptor, VEGF signal pathway, TNF signal pathway, NF-kappa B signaling pathway, fluid shear stress, atherosclerosis, etc. (Fig. 3A). Further analysis of the top 50 KEGG pathway gene sets of lymphatic endothelial cells revealed significant differences between groups in the VEGF pathway (Fig. 3B).

**Fig. 4:**
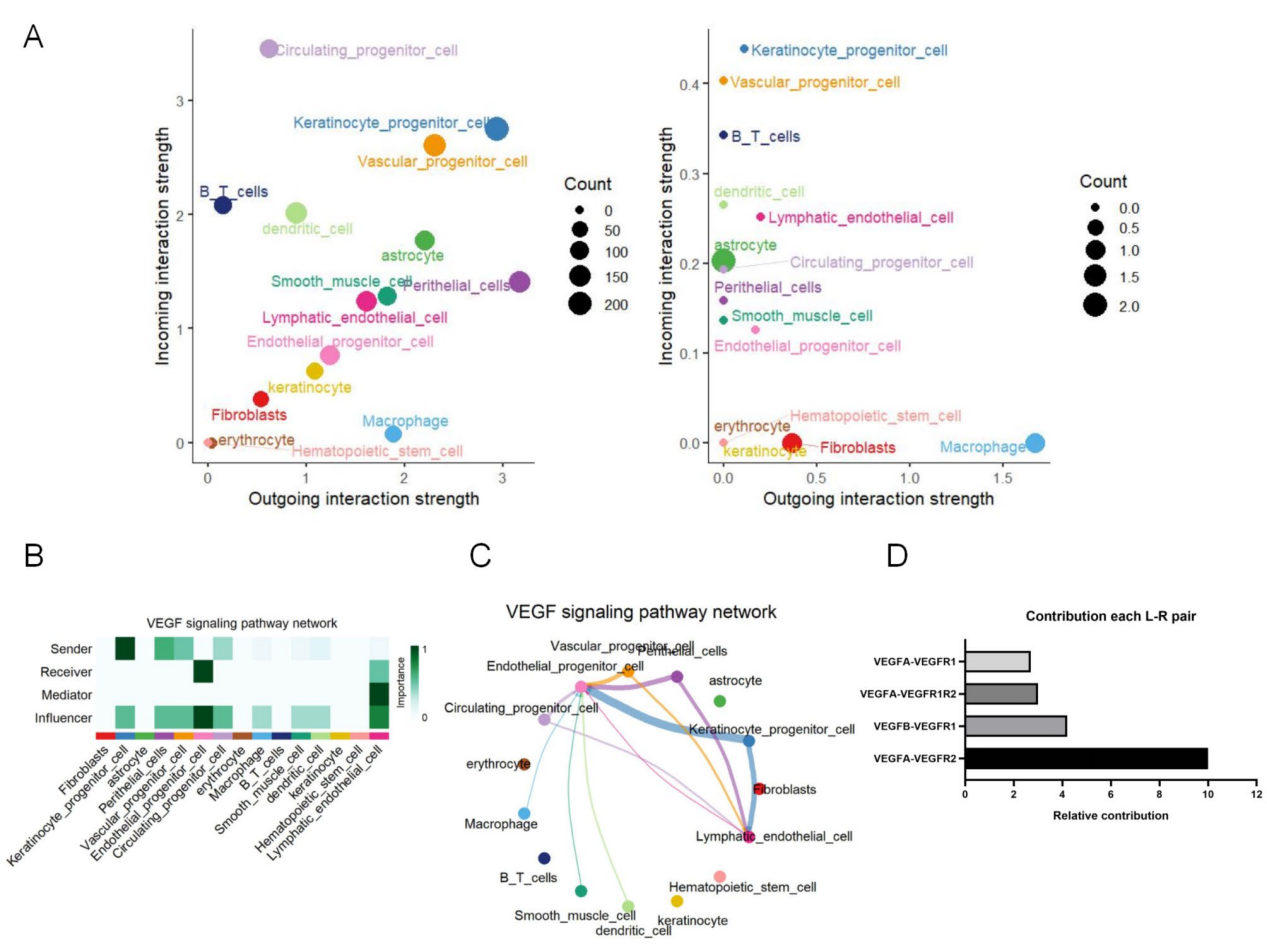
Lymphatic endothelial cells crosstalk with other cells through VEGF in CH. (**A**) Cellchat analysis reveals cellular communication in cell clusters. (**B**) To analyze the distribution of TGFβ signaling pathway in the gene-verse in cell communication. (**C**) Putative cell-cell interactions in the TGFβ signaling pathway (**D**). Correlation analysis of ligand-receptor pairs (VEGF and VEGF receptors) between the ligands secreted by lymphatic endothelial cell clusters

To determine the involvement of the VEGF signaling pathway in the abnormal expression of lymphatic endothelial cells in CH, we further analyzed the cell-cell communication between lymphatic endothelial cells and other cell clusters (Fig. 4A) and found that lymphatic endothelial cells are mainly regulated by other cells. Then, the regulation of VEGF signaling pathway in each cell cluster was analyzed, and it was found that keratinocyte progenitor cells were the main senders, endothelial progenitor cells were the main receivers and influencers, and lymphatic endothelial cells were the main mediators (Fig. 4B). By analyzing the VEGF signaling pathway network of various cell clusters, it was found that lymphatic endothelial cells are potentially regulated by vascular progenitor cells and endothelial perithelial cells (Fig. 4C). We next sought to elucidate the interactions between endothelial cells and other cell populations in the VEGF signaling pathway. Cellchat analysis revealed a marked increase in the interaction of receptor-ligand pairs between VEGFA and VEGFR2, suggesting tight cell-to-cell communication between lymphatic endothelial cells and other cell clusters via VEGFA-VEGFR2 (Fig. 4D).

### STAT1-KDR axis regulates VEGF signaling pathway in lymphatic endothelial cells in CH

**Fig. 5:**
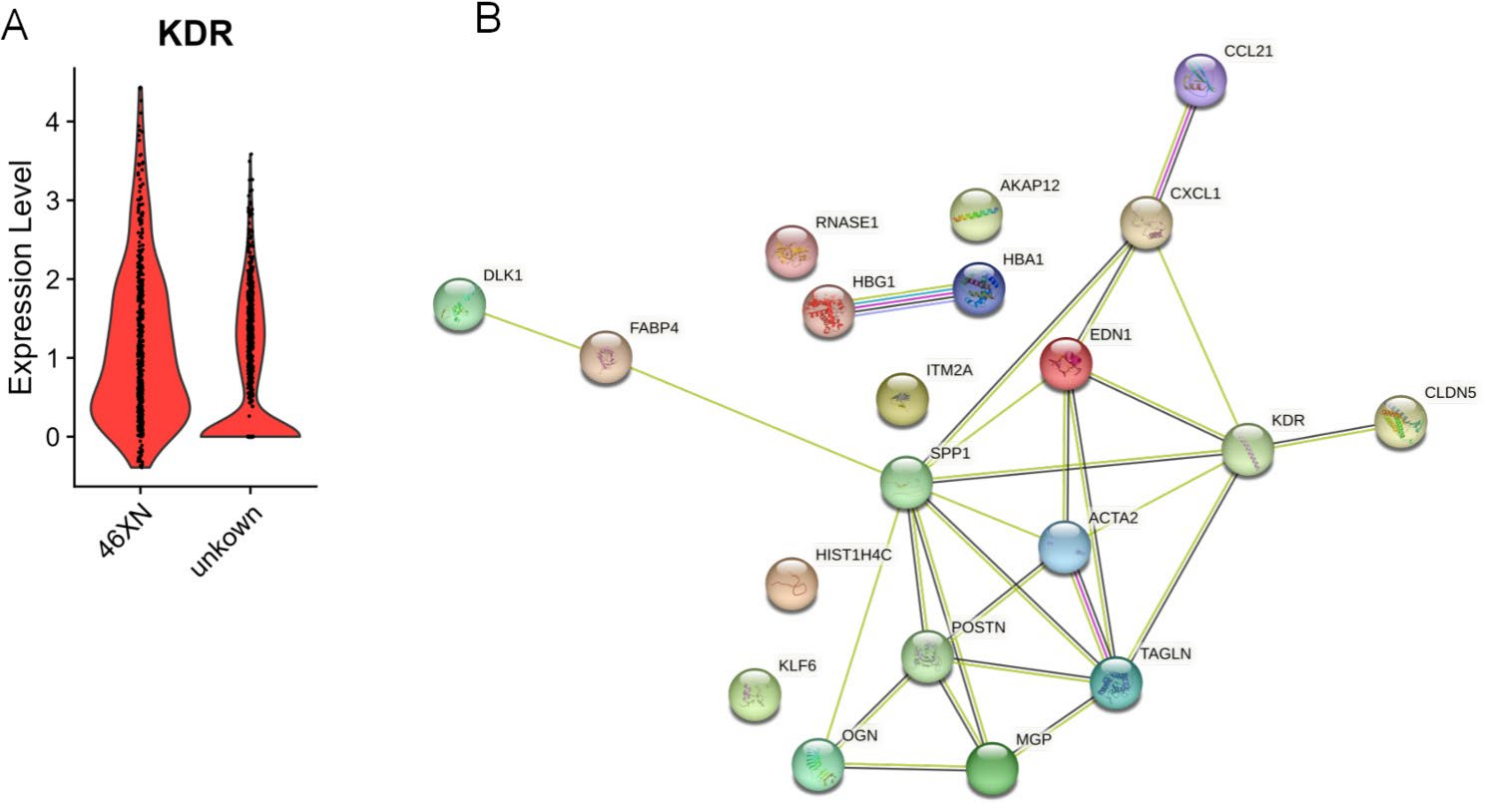
KDR is a key factor for the upregulation of VEGF signaling pathway in CH group. (**A**) The violin plot shows the differential expression of the gene KDR (VEGFR2 gene) in the VEGF signaling pathway between the CH group and the normal control group. (**B**) A simplified scheme showing protein interaction in the functional interaction network of VEGF signaling pathway. The interactions were generated using STRING analysis

Since VEGFA-VEGFR2 is abnormally enriched in lymphatic endothelial cells in lymphoid tissues of CH patients, we further analyzed the expression of VEGFR gene-*KDR*. As we expected, *KDR* was highly expressed in the lymphoid tissues of CH patients compared with normal controls (Fig. 5A). Additionally, PPI analysis with the differentially expressed genes of the lymphatic endothelial cell revealed that endothelin 1 (EDN1), actin-crosslinking protein (TAGLN), Claudin5 (CLDN5) is an important node, which has the possibility of cooperating with *KDR* gene to regulate VEGF pathway (Fig. 5B).

**Fig. 6:**
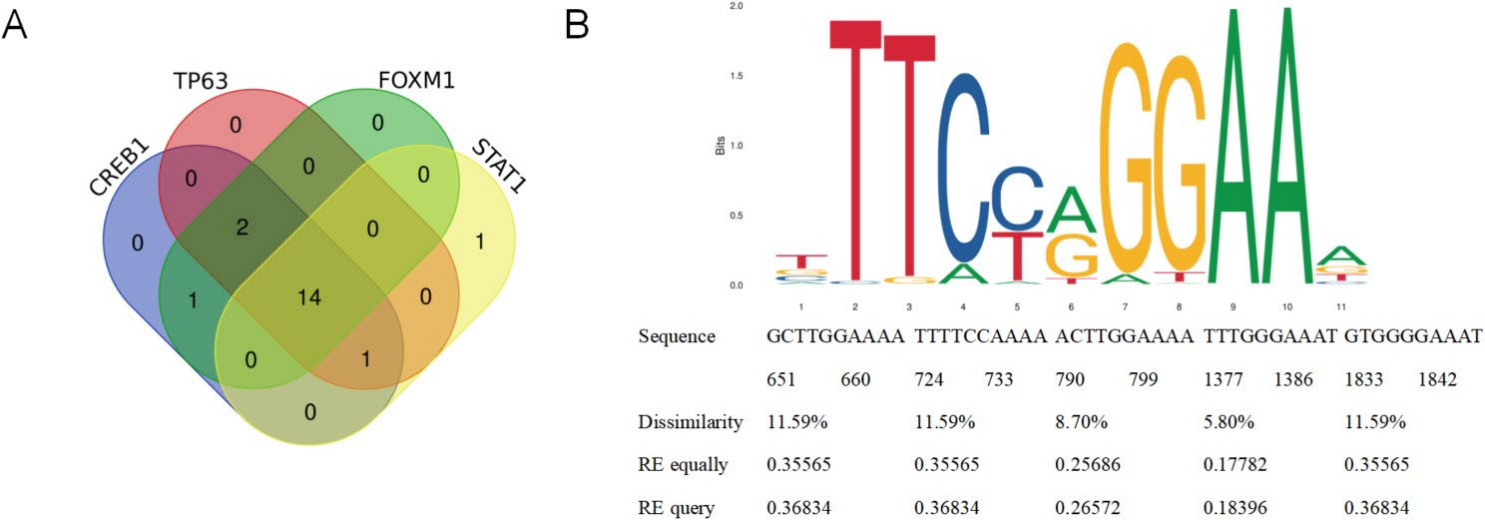
The transcription factor STAT1 may regulate the differential expression of KDR in CH. (**A**) Venn diagram showing genes predicted to be transcription factors of KDR and differentially expressed in CH group. (**B**) KnockTF predicted the binding region of KDR and STAT1 and the motif of STAT1 and KDR binding

To explore which of the differentially expressed genes can directly regulate the expression of *KDR* in lymphatic endothelial cells, we began to focus on the transcription factors that can regulate *KDR*. To further screen for transcription factors that regulate the *KDR* gene, first, we used KnockTF software to screen for transcription factors that are significantly enriched in lymphatic endothelial cells. Among them, CREB1, TP63, FOXM1, and STAT1 were found to be the transcription factors associated with *KDR* (top 4) (Fig. 6A). Furthermore, the *KDR* promoter transcription factor MOTIF (TGACG) was also predicted. As shown in Fig. 6B, the STAT1 transcription factor was found to bind to five binding sites in the *KDR* promoter region (Fig. 5B).

These results suggest that the transcription factor STAT1 may promote the expression of KDR to promote the proliferation of lymphatic endothelial cells and promote the exacerbation of CH.

## Discussion

In this paper, single-cell RNA sequencing technology was used to detect the difference in the proportion of 15 main cell clusters in lymphoid tissues of CH patients and healthy controls, and there were a large number of differentially expressed genes in each cell. Lymphatic endothelial cells, as a marker of abnormal proliferation of CH tumors, were abnormally increased in the CH tumor group. Functional enrichment of differential genes and analysis of cell-cell communication revealed that VEGF signaling pathway plays an important role in the regulation of CH in lymphatic endothelial cells. Finally, it was found that *KDR* in the VEGF signaling pathway was highly expressed in CH patients, and STAT1 may regulate the transcription of *KDR*.

Although the pathogenesis of CH is still unclear, it has been found that the main feature of CH patients is the abnormal proliferation of fibrous tissue among the lymphatic vessels [1, 18]. In this study, we found that fibroblasts accounted for the largest proportion of tumor tissue in CH patients, which may partially explain the abnormal proliferation of fibrous tissue in the middle of lymphatic vessels from the perspective of cell subsets. In skin cancer, mast cells can activate the proliferation programs of keratinocytes, endothelial cells, and fibroblasts by secreting angiogenesis-promoting factors and matrix remodeling-related enzymes [19] while the overexpression of VEGF-A/C/D in keratinocytes can promote and remodeling of lymphatic vessel formation [20]. In the present study, fibroblasts, keratinocytes, and endothelial progenitor cells are all increased in the tumor tissue of CH patients, which may be related to the activation of VEGF signaling pathway in the tumor microenvironment, and the increased keratinocytes further pass the positive feedback regulation promotes lymphatic vessel proliferation in CH patients.

Endothelial progenitor cells are the main source cells of lymphatic vessels under pathological conditions, and vascular progenitor cells and hematopoietic stem cells (HSCs) can be transformed into lymphatic endothelial progenitor cells [21]. Similarly, IECs can also be differentiated from vascular progenitor cells and HSCs [22–23]. Therefore, our results showed that vascular progenitor cells and HSCs were decreased and endothelial progenitor cells were increased in the tumor tissues of CH patients, which may be due to the conversion of HSCs and vascular progenitor cells into endothelial progenitor cells, which then differentiated into IECs.

The ligand-receptor composed of VEGF and VEGFR plays a key role in regulating the abnormal proliferation of lymphatic vessels [24–25]. VEGF receptors are highly expressed in both blood vessels and lymphatic vessels. VEGFR-1 is the highest in blood vessels, while VEGFR-2 is strongly expressed in lymphatic vessels. Specifically, vascular endothelial cells mainly express VEGFR-1 and − 2 while lymphatic endothelial cells express Cells predominantly express VEGFR-2 and − 3 [26]. The ligand family of VEGFR includes VEGF-A, -B, -C, -D, and placenta growth factor (PLGF) [27]. VEGF-A is the most potent inducer of endothelial response in the VEGF ligand family, which binds VEGFR-1 and VEGFR-2 [26]. Michael T. Dellinger et al. found that VEGFA promotes the proliferation and migration of LECs through VEGFR-2 rather than VEGFR-1 [28]. Although VEGF-C and VEGFR-3 can be used as clinicopathological features in other lymphomas (lymphangioma circumscriptum or intraabdominal lymphangioma), the distribution of VEGF-C in cystic hygroma is limited [29]. These reports are consistent with our single-cell RNA sequencing results, and our results also showed that VEGFA and VEGFR-2, a ligand-receptor pair, are enriched in lymphatic endothelial cells in CH patients.

In this study, we found that *KDR* was upregulated. *KDR* is the gene encoding vascular endothelial growth factor receptor VEGFR-2, which is one of the subtypes of VEGF receptors. In humans, VEGFR-2 is mainly expressed in lymphatic endothelial cells and plays a role in regulating endothelial cell proliferation by binding to its ligand VEGF [30]. KDR is overexpressed in neovascular tumor endothelial cells compared to normal endothelial cells [31]. KDR can promote endothelial cell proliferation and migration when activated by VEGF [32]. It has been demonstrated that KDR inhibitors have a potent anti-angiogenic effect in tumors. In addition, KDR was observed to be upregulated in breast cancer, colorectal cancer, and cell lymphoma [33]. In CH, the specific biological function and mechanism of KDR have not been reported in detail. We hypothesized that the significantly high expression of *KDR* in LECs suggests its important pathogenic role in CH. In addition, KDR acts as a signal transducer by binding to VEGF, this further provides evidence that KDR plays an important role in CH.

Evidence has shown that the transcription of *KDR* is regulated by transcription factors [34]. Moreover, endothelial function is also regulated by many transcription factors, such as AP-1 [35] and zinc finger transcription factors [36]. However, the possible transcription factors that potentially regulate KDR in CH remain unclear. Our bioinformatics prediction results showed that the STAT1 transcription factor could bind to the *KDR* promoter region. STAT1 plays an important role in cardiovascular diseases. STAT1 overexpression promotes endothelial cell injury and inflammation. Overexpression of the transcription factor STAT1 has been found to promote endothelial cell injury and inflammation [37–39]. Therefore, the predicted binding of STAT1 and KDR may be related to *KDR* overexpression and endothelial dysfunction in CH, which needs further investigation. We will continue to collect samples from CH patients and validate our single-cell sequencing results using qPCR, western blotting, immunohistochemistry, flow cytometry, and other experimental methods.

## Conclusion

In the present study, we found that VEGF can regulate CH by regulating the communication between lymphatic endothelial cell clusters and other cells through single-cell RNA sequencing, and analyzed the potential of STAT1 to promote *KDR* transcriptional regulation of the VEGF signaling pathway. The limitation of this article is that the number of clinical samples included in our study is limited, and it is impossible to comprehensively explain the pathological characteristics of all CH patients. Our results suggest a potentially important role of endothelial KDR in CH pathogenesis, which needs to be verified by subsequent experiments. Since a detailed understanding of the pathogenesis of CH is still lacking, our findings provide some clues to the pathogenesis of CH from the perspective of cell subsets.

### Electronic supplementary material

Below is the link to the electronic supplementary material.

Supplementary Material 1

## Data Availability

The original contributions presented in the study are included in the article, further inquiries can be directed to the corresponding author.
